# A Case of Lafora Disease Diagnosed by Axillary Skin Biopsy*

**DOI:** 10.5146/tjpath.2021.01522

**Published:** 2021-09-15

**Authors:** Elife Kımıloğlu, Pelin AKBAŞ, Özgül Esen Öre, Çağla Turan

**Affiliations:** Department of Pathology, Health Sciences University, Istanbul Gaziosmanpasa Research and Training Hospital, İstanbul, Turkey; Department of Neurology, Health Sciences University, Istanbul Gaziosmanpasa Research and Training Hospital, İstanbul, Turkey

**Keywords:** Lafora disease, Skin biopsy, Epilepsy

## Abstract

Lafora disease is a severe form of progressive myoclonic epilepsy with autosomal recessive inheritance diagnosed by inclusion body in biopsy. A 26-year-old woman was admitted due to complaints of frequent twitches and fainting. The 0.5x0.3x0.3 cm axillary skin punch biopsy was subjected to routine histopathological evaluation. Cytoplasmic PAS-positive inclusion bodies were observed at the basal side of the eccrine and apocrine glands. The diagnosis of Lafora disease can also be made by the observation of the polyglycosan cytoplasmic inclusion bodies in the brain, liver and skeletal muscle biopsies. Although we need more work to understand the etiopathogenesis of Lafora disease, we would like to draw attention to the importance of skin biopsy in the differential diagnosis of young patients with clinically refractory epilepsy, myoclonus, and cognitive decline.

## INTRODUCTION

Lafora disease is a rare, autosomal recessive severe form of progressive myoclonic epilepsy characterized by myoclonus, focal-generalized seizures, and progressive dementia (1–3). The presence of Periodic Acid-Schiff stain positive inclusion bodies is diagnostic for Lafora disease (1,3,4). In this article, we present and discuss the case of a 26-year-old female patient who presented with the complaints of contractions all over her body, increasingly frequent twitches in the eyes and shoulders for the last 2-3 days, and frequent fainting.

## CASE REPORT

**Figure 1:**
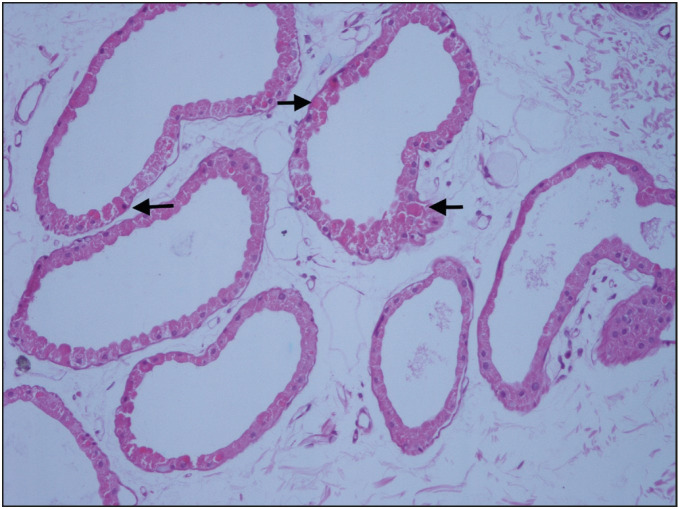
Biopsy from axillary region shows intracytoplasmic spherical inclusions (arrows) in the sweat gland duct (H&E; 200).

**Figure 2:**
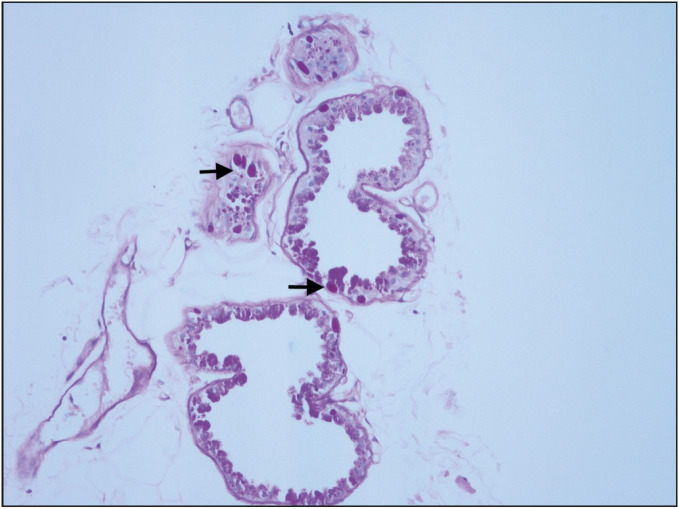
PAS positive inclusion bodies (arrows) in apocrine glands (PAS; x200).

A 26-year-old woman was admitted due to complaints of frequent twitches and fainting. She had experienced these gradually worsening twitches for 11 years, she had repeated every academic year twice until the third year of school, she could not learn how to read or write, her parents were relatives, and her brother had died at the age of 37 and had also suffered from epilepsy. She was currently taking 1500 mg/day valproic acid, 200 mg/day phenytoin, and 1000 mg/day levetiracetam for seizure control. She was not using her medication regularly. Her neurological examination showed frequent twitches in the eyelids, shoulders and feet. Cerebellar tests could not be evaluated due to bilateral myoclonus. In the EEG, severe generalized disorganization was observed with generalized spike wave discharges accompanied by myoclonus. The skin was biopsied and sent to the pathology laboratory to find the etiology of this persistent, medication-resistant seizure disorder. The 0.5x0.3x0.3 cm axillary skin punch biopsy was subjected to routine histopathological evaluation. No histopathological abnormalities were observed when the paraffin-embedded sections were stained with H&E, but staining with Periodic Acid-Schiff revealed cytoplasmic PAS-positive inclusion bodies at the basal side of the eccrine and apocrine glands (Figure 1 Figure 2).

## DISCUSSION

Lafora bodies seen in light microscopy of skin biopsy when taken in accordance with appropriate clinical data are diagnostic for Lafora Disease. The presence of Lafora bodies alone, independent of the clinical context, is not diagnostic (1). As discussed earlier, similar appearances can also be seen in other diseases and some physiological bodily processes. These bodies may be seen in diseases such as double athetosis, ALS, type 4 glycogen depot disease, and adult polyglycosan body disease, whereas similar polyglycosan bodies can also be seen in the normal aging process as corpora amylacea (5). Therefore, current histopathologic data should not be evaluated independent of the clinical context. The PAS positive staining of these bodies, as we have shown in our case, indicates that they are carbohydrate-rich at significant levels (3,4,6,7). Former electron microscopic evaluations have shown that the Lafora body is almost entirely composed of complex glucose molecules, indicating that the carbohydrate content is almost entirely polyglycosan (glucose polysaccharides) (1,8). EPM2A (6q24), EPM2B (6q22.3) and PRDM8 (shown only in one family) are said to be responsible for the mutation that cause these polyglycosans to accumulate (2). The diagnosis of Lafora disease can also be made by the observation of the polyglycosan cytoplasmic inclusion bodies in the brain, liver, and skeletal muscle biopsies (1,5,8). On the other hand, polyglycosan bodies seen in Lafora disease are separated from other diseases in which polyglycosan bodies are also observed, by the location of accumulation. In Lafora disease, polyglycosan bodies are concentrated on the perikaryon and dendrites area of the neuron while in adult polyglycosan body disease and corpora amylacea, the polyglycosan bodies are limited to the axonal region (1). Why they do not proceed along the same path as the other polyglycosans is curious in Lafora Disease. Although we need more work to understand the etiopathogenesis of Lafora disease, we would like to draw attention to the importance of skin biopsy in the differential diagnosis of young patients with clinically refractory epilepsy, myoclonus, and cognitive decline.

## Conflict of INTEREST

The authors declare no conflict of interest.

## References

[ref-1] Minassian B. A. (2001). Lafora's disease: towards a clinical, pathologic, and molecular synthesis. Pediatr Neurol.

[ref-2] Baykan Betul, Striano Pasquale, Gianotti Stefania, Bebek Nerses, Gennaro Elena, Gurses Candan, Zara Federico (2005). Late-onset and slow-progressing Lafora disease in four siblings with EPM2B mutation. Epilepsia.

[ref-3] White J. W., Gomez M. R. (1988). Diagnosis of Lafora disease by skin biopsy. J Cutan Pathol.

[ref-4] Karimipour D., Lowe L., Blaivas M., Sachs D., Johnson T. M. (1999). Lafora disease: diagnosis by skin biopsy. J Am Acad Dermatol.

[ref-5] Turnbull Julie, Tiberia Erica, Striano Pasquale, Genton Pierre, Carpenter Stirling, Ackerley Cameron A., Minassian Berge A. (2016). Lafora disease. Epileptic Disord.

[ref-6] Rubio G., Garcia Guijo C., Mallada J. J., Cabello A., Garcia Merino A. (1992). Diagnosis by axilla skin biopsy in an early case of Lafora's disease. J Neurol Neurosurg Psychiatry.

[ref-7] Sathiah Prasath, Gochhait Debasis, Dehuri Priyadarshini, Subramanian Hema (2017). Diagnosis of Lafora Disease by Skin Biopsy. J Clin Diagn Res.

[ref-8] Ceuterick C., Martin J. J. (1984). Diagnostic role of skin or conjunctival biopsies in neurological disorders. An update. J Neurol Sci.

